# Understanding differences within ethnic group designation: comparing risk factors and health indicators between Iranian and Arab Americans in Northern California

**DOI:** 10.1186/s12889-021-11121-z

**Published:** 2021-06-05

**Authors:** Nadia N. Abuelezam, Abdulrahman El-Sayed, Sandro Galea, Nancy P. Gordon

**Affiliations:** 1 Boston College William F. Connell School of Nursing, 140 Commonwealth Avenue, Chestnut Hill, MA 02467 USA; 2 College of Literature, Science, & the Arts & Department of Internal Medicine, University of Michigan, Ann Arbor, MI USA; 3 Boston University School of Public Health, Boston, MA USA; 4 Kaiser Permanente Division of Research, Oakland, CA USA

**Keywords:** Health disparities, Epidemiology, Electronic health records, Ethnicity, Iranian Americans, Arab Americans

## Abstract

**Background:**

The Middle Eastern and North African (MENA) ethnic group is a diverse group composed of two primary subsets in the United States: Iranian and Arab Americans. We aimed to compare health risk factors, chronic health conditions, and mental health conditions of Iranian and Arab American adults in Northern California.

**Methods:**

We used cross-sectional electronic health record (EHR) data from a 2016 Northern California health plan study cohort to compare adults classified as Iranian or Arab American based on ethnicity, language, or surname. We produced age-standardized prevalence estimates of obesity, smoking, hyperlipidemia, prediabetes, diabetes, hypertension, depression, and anxiety for Iranian and Arab American men and women by age group (35–44, 45–64, and 65–84) and overall (35–84). We used generalized linear models to calculate prevalence ratios (PR) to compare Iranian and Arab American adults ages 35–84 on all health indicators.

**Results:**

Compared to Arab Americans, Iranian Americans had a lower prevalence of obesity (PR: 0.77, 95% confidence interval, CI: 0.73, 0.82), current smoking (PR: 0.80, 95% CI: 0.73, 0.89), and ever smoking (PR: 0.95, 95% CI: 0.91, 0.99), but a higher prevalence of hyperlipidemia (PR: 1.09, 95% CI: 1.06, 1.12), prediabetes (PR: 1.12, 95% CI: 1.09, 1.16), depression (PR; 1.41, 95% CI: 1.30, 1.52), and anxiety (PR: 1.52, 95% CI: 1.42, 1.63). Similar patterns were observed for men and women.

**Conclusion:**

This work supports the need to collect granular data on race and ethnicity within the MENA ethnic group to improve identification in clinical care settings and population health reporting to better address the physical and mental health needs of different MENA subgroups.

## Introduction

The Middle Eastern and North African ethnic category (MENA) is diverse and is composed of two primary groups in the United States (US): Arab Americans and Iranian Americans. Arab Americans have cultural or ethnic origins in one of 22 Arabic speaking countries in the Middle East and North Africa. Arab Americans have been consistently immigrating to the US in multiple migration waves since the 1880s. There are approximately 4 million Arab Americans living in the US, with the largest number residing in California [1]. The other large subset in the MENA designation are Iranian Americans. The majority of the approximately 0.5–1 million Iranian Americans identify as first- or second- generation Iranian immigrants, and live in California [2]. Initial Arab immigrants to the US were primarily Christian, although recent waves are more commonly majority Muslim. Iranian immigrants are most commonly Muslim, with a small minority participating in other religions. The start of Iranian immigration to the US began shortly after the 1979 Revolution. Those who left Iran after the revolution were adults and professionals [2], making the circumstances of their immigration different from Arab immigration patterns [3]. Both Arab Americans and Iranian Americans make up the majority of the MENA ethnic category in the US, but little is known about potential differences in health indicators between these two groups.

Within ethnic categories, differences in risk factors and health indicators have been observed for Latinx, Asian, and Black Americans. While some differences within ethnic categories have been attributed to country of origin [4–6], others are related to differences in lived experiences in the US which result from differential experiences with discrimination and stigma [7–9]. Research into health differences within ethnic categories has also led to observations about immigration related health patterns, like the healthy immigrant paradox, which does not seem to hold for all ethnic groups [10–14]. While the majority of research in this area has focused on racial and ethnic minority groups, recent evidence suggests there is substantial diversity within the non-Hispanic White category on health measures found in the American Community Survey, which includes MENA Americans [15].

No studies, to our knowledge, have attempted to identify potential differences in health characteristics between groups within the MENA ethnic category. To address this knowledge gap, we used electronic health record (EHR) data from a large health care delivery system in Northern California to compare risk factors, chronic and mental health conditions of Arab Americans and Iranian Americans who were health plan members in this system during the 2016 calendar year. Our aim was to document similarities and differences in the health-related profiles of Arab and Iranian Americans to raise awareness about the health needs of each subgroup, including priorities for primary and secondary prevention.

## Methods

### Setting and study sample

This study used data for 18,072 Arab Americans and 5777 Iranian Americans who were identified from a 2016 race/ethnicity study cohort of Kaiser Permanent North California (KPNC) members aged 35–84. KPNC is an integrated health care delivery system that provides primary and specialty healthcare, pharmacy, and laboratory services to over 3.4 million adults in the San Francisco and Greater Bay Area. Details about the EHR study cohort and KPNC can be found elsewhere [16]. This study was approved by Kaiser Permanente Northern California’s Institutional Review Board.

### Ethnicity

Ethnicity was determined from EHR data using a combination of strategies. First, race and ethnic codes assigned individuals coded as Iranian or Persian to the Iranian American group and individuals with a code for any of 22 Arab League countries to the Arab American group. Second, for those not assignable this way, we used preferred spoken and written language indicated in the EHR. Farsi speakers (who did not have an Afghani ethnicity code or a recognizable Afghani surname) were assigned to the Iranian American group and Arabic speakers were assigned to the Arab American group. As a last step, we used a vetted Arab surname list to identify likely Arab Americans [17].

### Outcomes of interest

Outcomes were divided into three groups: risk factors, chronic health conditions, and mental health conditions. All outcomes of interest were ascertained from EHR and disease registry data as described in Gordon et al. [16]. Risk factors included obesity, current and ever smoking, hyperlipidemia diagnosis, and prediabetes diagnosis. Chronic health conditions included diagnosed diabetes and hypertension. Mental health conditions included diagnosed depression and anxiety. Obesity (BMI ≥ 30 kg/m^2^) was calculated using height and weight data recorded closest to December 1, 2016. Current and ever smoking were determined from tobacco use data closest to December 1, 2016, going back up to 2012 for individuals who did not have more recent data or were only recorded as nonsmokers. Hyperlipidemia, hypertension, depression, and anxiety were determined from ICD-9 and ICD-10 ambulatory visit diagnosis codes in the individual’s EHR during calendar years 2015–2016 or from the December 2016 problem list. Diabetes was determined from presence in the health plan’s Diabetes Registry by December 31, 2016. Prediabetes was determined by having an ICD-9 or ICD-10 visit code for prediabetes or a 2016 lab value in the prediabetes range (HgA1c in the 5.7–6.4 range or fasting glucose in the 100- < 126 range) and not being in the Diabetes Registry by December 31, 2016. For more details, see Gordon et al. [16].

### Analysis

Data were analyzed using SAS version 9.4 (SAS Institute, Cary, IN, 2013). To control for differences in the age compositions of the Arab and Iranian American groups, we used a Proc Surveyreg procedure recommended by the Centers for Disease Control and Prevention [18] to produce age-standardized prevalence estimates for these groups, overall and by sex. Age-standardized prevalence estimates were similarly calculated for three age subgroups (35–44, 45–64, 65–84 years of age). We used 2016 US Census counts for five age groups (35–44, 45–54, 55–64, 65–75, and 75–84) to produce age-standardized prevalence estimates for the 35–84 age group and two age groups (45–54, 55–64 and 65–74, 75–84) to produce estimates for the 45–64 and 65–84 age groups, respectively. Proc Genmod was used to calculate sex-stratified and age-adjusted prevalence ratios (PR) for Iranian Americans compared to Arab Americans. Statistically significant differences mentioned in the text are at the *p* < 0.05 level.

## Results

### Sample characteristics

The Arab American and Iranian American women in the study cohort were similar in age, with mean ages of 53.2 years and 55.7 years, respectively. Among men, the Arab American group was significantly younger than the Iranian Arab American group (mean ages of 53.2 years and 59.0 years, respectively). Arab American men had greater representation in the younger age group than Iranian American men with 29.4% versus 14.2% in the 35–44 age group and 18.7% versus 36.4% in the 65–84 age group, respectively. Iranian American women had a younger age distribution than Iranian American men, with 23.9% of women and 14.2% of men in the 35–44 age group; Arab American women and men had similar age distributions. While both groups were majority English speakers, adults in the Iranian American group were less likely than those in the Arab American group to have English indicated as their spoken language (87.0% versus 95.4%, *p* < 0.01).

### Risk factors

Across all age groups, Arab American men and women had a higher prevalence of obesity than Iranian American men and women, with the highest prevalence among all groups found in the 45–64 age group (Table 1). Prevalence of current smoking was higher among Arab American than Iranian American men and women ages 35–84, but within age groups, the difference was only significant among women aged 35–44. In the 35–84, 45–64 and 65–84 age groups, Iranian American women and men had a higher prevalence of diagnosed hyperlipidemia and prediabetes than Arab American women and men. Iranian Americans had a lower overall age-adjusted prevalence of obesity (PR: 0.77, 95% CI: 0.73, 0.82) and current smoking (PR: 0.80, 95% CI: 0.73, 0.89) and higher overall prevalence of hyperlipidemia (PR: 1.09, 95% CI: 1.06 1.12) and prediabetes (PR: 1.12, 95% CI: 1.09, 1.16) than Arab Americans (Table 2); these patterns were seen in both men and women.

**Table 1:** Prevalence of chronic disease risk factors, chronic health, and mental health outcomes stratified by Arab or Iranian ethnicity, sex, and age group from a Northern California health system in 2016

	All Iranian Americans	All Arab Americans	Iranian American Men	Arab American Men	Iranian American Women	Arab American Women
	Prevalence (95% CI)	Prevalence (95% CI)	Prevalence (95% CI)	Prevalence (95% CI)	Prevalence (95% CI)	Prevalence (95% CI)
**Ages 35–84**	(*N* = 5777)	(*N* = 18,072)	(*N* = 2931)	(*N* = 9353)	(*N* = 2846)	(*N* = 8719)
*Risk factors*						
Obesity (BMI > =30)	25.1 (23.8, 26.4)	32.8 (32.0, 33.6) ^a^	27.8 (25.8, 29.8)	34.4 (33.2, 35.5) ^a^	22.9 (21.1, 24.7)	31.0 (29.9, 32.2) ^a^
Current Smoking	7.7 (7.0, 8.4)	10.0 (9.5, 10.4) ^a^	10.9 (9.6, 12.1)	13.3 (12.6, 14.0) ^a^	5.1 (4.3, 5.9)	6.5 (5.9, 7.0) ^a^
Ever Smoking	30.8 (29.6, 32.0)	33.4 (32.7, 34.1) ^a^	39.7 (37.8, 41.5)	41.8 (40.7, 42.8)	22.2 (20.7, 23.7)	24.8 (23.9, 25.7) ^a^
Hyperlipidemia	40.3 (39.2, 41.4)	36.3 (35.7, 37.0) ^a^	45.2 (43.5, 46.9)	40.8 (39.8, 41.7) ^a^	35.9 (34.4, 37.4)	31.5 (30.6, 32.4) ^a^
Prediabetes	33.4 (32.2, 34.6)	27.7 (27.0, 28.4) ^a^	35.9 (34.1, 37.7)	31.0 (30.0, 32.1) ^a^	31.1 (29.4, 32.8)	24.4 (23.4, 25.3) ^a^
*Chronic health conditions*						
Diabetes	14.1 (13.2, 14.9)	14.3 (13.7, 14.8)	16.2 (15.0, 17.4)	17.3 (16.6, 18.1)	11.6 (10.5, 12.7)	11.1 (10.4, 11.7)
Hypertension	26.7 (25.7, 27.6)	28.2 (27.6, 28.8) ^a^	29.1 (27.7, 30.6)	30.5 (29.6, 31.3)	24.2 (22.8, 25.5)	25.8 (25.0, 26.7)
*Mental health conditions*						
Depression	12.7 (11.9, 13.6)	9.1 (8.6, 9.5) ^a^	7.8 (6.8, 8.8)	6.1 (5.6, 6.6) ^a^	17.7 (16.4, 19.1)	12.1 (11.4, 12.8) ^a^
Anxiety	16.6 (15.6, 17.6)	10.9 (10.4, 11.4) ^a^	11.7 (10.5, 13.0)	8.1 (7.5, 8.7) ^a^	21.7 (20.2, 23.2)	13.8 (13.1, 14.6) ^a^
**Ages 35–44**	(*N* = 1098, 19.0%)	(*N* = 5243, 29.0%)	(*N* = 417, 14.2%)	(*N* = 2753, 29.4%)	(*N* = 681, 23.9%)	(*N* = 2490, 28.6%)
*Risk factors*						
Obesity (BMI > =30)	22.6 (19.6, 25.6)	30.8 (29.2, 32.3) ^a^	26.5 (21.5, 31.6)	34.7 (32.5, 37.0) ^a^	20.2 (16.6, 23.8)	26.7 (24.6, 28.7) ^a^
Current Smoking	7.1 (5.6, 8.7)	11.8 (10.9, 12.7) ^a^	13.3 (9.9, 16.7)	17.0 (15.6, 18.5)	3.5 (2.2, 4.9)	6.4 (5.5, 7.4) ^a^
Ever Smoking	19.0 (16.7, 21.4)	27.3 (26.0, 28.5) ^a^	28.6 (24.2, 33.1)	36.7 (34.8, 38.5) ^a^	20.2 (16.6, 23.8)	17.5 (16.0, 19.0)
Hyperlipidemia	10.7 (8.9, 12.6)	10.6 (9.8, 11.5)	16.3 (12.8, 19.9)	15.1 (13.8, 16.5)	7.3 (5.4, 9.3)	5.7 (4.8, 6.6)
Prediabetes	12.1 (10.1, 14.0)	11.5 (10.6, 12.4)	14.7 (11.2, 18.1)	13.5 (12.2, 14.8)	10.5 (8.2, 12.8)	9.3 (8.2, 10.5)
*Chronic health conditions*						
Diabetes	3.0 (2.0, 4.0)	3.6 (3.1, 4.2)	3.8 (2.0, 5.7)	4.7 (3.9, 5.5)	2.5 (1.3, 3.7)	2.5 (1.9, 3.1)
Hypertension	4.3 (3.1, 5.5)	5.8 (5.2, 6.5)	5.8 (3.5, 8.0)	7.0 (6.0, 7.9)	3.4 (2.0, 4.7)	4.5 (3.7, 5.4)
*Mental health conditions*						
Depression	10.5 (8.7, 12.3)	7.6 (6.9, 8.3) ^a^	6.5 (4.1, 8.8)	4.8 (4.0, 5.6)	12.9 (10.4, 15.4)	10.6 (9.4, 11.8)
Anxiety	17.2 (15.0, 19.4)	11.3 (10.4, 12.1) ^a^	12.7 (9.5, 15.9)	8.4 (7.4, 9.4) ^a^	20.0 (17.0, 23.0)	14.5 (13.1, 15.8) ^a^
**Ages 45–64**	(*N* = 2909, 50.4%)	(*N* = 9214, 51.0%)	(*N* = 1513, 51.6%)	(*N* = 4847, 51.8%)	(*N* = 1396, 49.1%)	(*N* = 4367, 50.1%)
*Risk factors*						
Obesity (BMI > =30)	26.7 (24.8, 28.5)	34.5 (33.3, 35.6) ^a^	30.7 (27.7, 33.4)	36.7 (35.1, 38.3) ^a^	22.5 (20.0, 25.0)	32.1 (30.5, 33.7) ^a^
Current Smoking	9.1 (8.0, 10.1)	11.0 (10.4, 11.7) ^a^	11.9 (10.2, 13.6)	14.1 (13.1, 15.2)	6.2 (4.9, 7.5)	7.6 (6.8, 8.4)
Ever Smoking	32.0 (30.3, 33.6)	32.4 (31.4, 33.4)	40.3 (37.7, 42.8)	39.3 (37.9, 40.7)	23.7 (21.5, 25.9)	24.9 (23.6, 26.2)
Hyperlipidemia	38.6 (36.9, 40.3)	33.6 (32.6, 34.5) ^a^	45.2 (42.7, 47.7)	39.3 (37.9, 40.6) ^a^	32.0 (29.7, 34.3)	27.3 (26.0, 28.6) ^a^
Prediabetes	33.4 (31.6, 35.2)	26.9 (26.0, 27.9) ^a^	35.6 (33.0, 38.2)	30.5 (29.1, 31.9) ^a^	31.5 (29.0, 34.0)	23.3 (22.0, 24.6) ^a^
*Chronic health conditions*						
Diabetes	12.0 (10.8, 13.1)	12.7 (12.0, 13.4)	14.7 (13.0, 16.5)	15.8 (14.7, 16.8)	8.8 (7.3, 10.3)	9.3 (8.4, 10.2)
Hypertension	20.9 (19.4, 22.3)	23.4 (22.5, 24.2) ^a^	24.8 (22.7, 27.0)	25.8 (24.6, 27.0)	16.7 (14.8, 18.6)	20.6 (19.4, 21.7) ^a^
*Mental health conditions*						
Depression	11.9 (10.7, 13.1)	8.4 (7.8, 8.9) ^a^	7.1 (5.8, 8.4)	5.7 (5.1, 6.4)	16.8 (14.8, 18.8)	11.3 (10.4, 12.3) ^a^
Anxiety	15.8 (14.5, 17.1)	10.5 (9.8, 11.1) ^a^	11.4 (9.9, 13.0)	8.2 (7.4, 8.9) ^a^	20.4 (18.3, 22.6)	13.0 (12.0, 14.0) ^a^
**Ages 65–84**	(*N* = 1770, 30.6%)	(*N* = 3615, 20.0%)	(*N* = 1001, 34.2%)	(*N* = 1753, 18.7%)	(*N* = 769, 27.0%)	(*N* = 1862, 21.3%)
*Risk factors*						
Obesity (BMI > =30)	24.5 (22.3, 26.8)	31.3 (29.6, 33.1) ^a^	23.3 (20.3, 26.2)	29.5 (27.1, 31.9) ^a^	26.2 (22.6, 29.8)	33.1 (30.7, 35.6) ^a^
Current Smoking	5.6 (4.5, 6.7)	6.1 (5.3, 6.9)	6.5 (5.0, 8.0)	8.1 (6.8, 9.4)	4.4 (3.0, 5.9)	4.3 (3.3, 5.2)
Ever Smoking	39.7 (37.4, 41.9)	41.2 (39.6, 42.8)	48.9 (45.8, 52.0)	51.4 (49.1, 53.8)	27.6 (24.5, 30.7)	31.7 (29.5, 33.8)
Hyperlipidemia	71.6 (69.5, 73.7)	65.9 (64.4, 67.4) ^a^	72.6 (69.8, 75.3)	67.9 (65.8, 70.1) ^a^	70.4 (67.2, 73.6)	64.0 (61.9, 66.2) ^a^
Prediabetes	53.5 (50.9, 56.2)	44.5 (42.7, 46.3) ^a^	56.6 (53.1, 60.1)	48.8 (46.1, 51.4) ^a^	49.7 (45.8, 53.7)	40.8 (38.4, 43.3) ^a^
*Chronic health conditions*						
Diabetes	28.6 (26.5, 30.7)	27.3 (25.9, 28.8)	30.8 (28.0, 33.6)	32.4 (30.2, 34.6)	25.8 (22.7, 28.9)	22.6 (20.7, 24.5)
Hypertension	59.3 (57.0, 61.5)	58.9 (57.4, 60.5)	59.8 (56.8, 62.8)	61.7 (59.5, 64.0)	58.6 (55.2, 62.0)	56.3 (54.1, 58.5)
*Mental health conditions*						
Depression	16.4 (14.7, 18.2)	11.8 (10.8, 12.9) ^a^	10.5 (8.6, 12.4)	8.2 (6.9, 9.5)	24.2 (21.2, 27.2)	15.2 (13.6, 16.9) ^a^
Anxiety	17.7 (15.9, 19.5)	11.4 (10.3, 12.4) ^a^	11.4 (9.4, 13.4)	7.7 (6.4, 8.9) ^a^	25.9 (22.8, 29.0)	14.8 (13.2, 16.4) ^a^

^a^ Significant (*p* < 0.01) difference between Iranian and Arab Americans

**Table 2:** Age-adjusted prevalence ratios comparing Iranian Americans to Arab Americans ages 35–84 on risk factors, chronic health, and mental health outcomes

	PR (95% CI)
**Overall**	
*Risk factors*	
Obesity	0.77 (0.73, 0.82)
Ever smoking	0.95 (0.91, 0.99)
Current smoking	0.80 (0.73, 0.89)
Hyperlipidemia	1.09 (1.06, 1.12)
Prediabetes	1.12 (1.09, 1.16)
*Chronic health conditions*	
Diabetes	0.98 (0.92, 1.05)
Hypertension	0.97 (0.93, 1.00)
*Mental health conditions*	
Depression	1.41 (1.30, 1.52)
Anxiety	1.52 (1.42, 1.63)
**Men**	
*Risk factors*	
Obesity	0.81 (0.75, 0.88)
Ever smoking	0.97 (0.92, 1.02)
Current smoking	0.82 (0.73, 0.93)
Hyperlipidemia	1.10 (1.06, 1.14)
Prediabetes	1.16 (1.09, 1.23)
*Chronic health conditions*	
Diabetes	0.95 (0.87, 1.03)
Hypertension	0.97 (0.92, 1.02)
*Mental health conditions*	
Depression	1.28 (1.10, 1.48)
Anxiety	1.46 (1.29, 1.65)
**Women**	
*Risk factors*	
Obesity	0.74 (0.68, 0.80)
Ever smoking	0.91 (0.84, 0.98)
Current smoking	0.79 (0.66, 0.94)
Hyperlipidemia	1.13 (1.07, 1.18)
Prediabetes	1.27 (1.19, 1.36)
*Chronic health conditions*	
Diabetes	1.06 (0.95, 1.19)
Hypertension	0.98 (0.92, 1.04)
*Mental health conditions*	
Depression	1.46 (1.33, 1.61)
Anxiety	1.57 (1.44, 1.71)

*PR* prevalence ratio, *CI* confidence interval

### Chronic health conditions

Among both Iranian and Arab Americans, men in the 45–64 and 65–84 year olds had a higher prevalence of diabetes than women, and in the 45–64 age group, a higher prevalence of hypertension (Table 1). Across all age groups, the prevalence of diabetes within gender groups was similar for Iranian and Arab Americans. In the 45–64 age group, the prevalence of hypertension was lower in Iranian American women than Arab American women (16.7% vs. 20.6%). Iranian Americans aged 35–84 had a lower overall age-adjusted prevalence of hypertension (PR: 0.97, 95% CI: 0.93, 1.00) than Arab Americans, with similar patterns seen among men and women (Table 2).

### Mental health conditions

Across all age groups, prevalence of clinically diagnosed anxiety was higher among Iranian American men and women compared to Arab Americans (Table 1). Prevalence of clinically diagnosed depression was higher among Iranian American women than Arab American women in all age groups, with no significant ethnic group differences among men. Notably, in both ethnic groups, prevalence of diagnosed depression was higher among women and men in the 65–84 age group than in the other age groups. Older Iranian American women had the highest burden of mental health issues, with approximately 26 and 24% having diagnoses of anxiety and depression, respectively. Iranian Americans had higher overall age-adjusted prevalence of diagnosed anxiety (PR: 1.52, 95% CI: 1.42, 1.63) and depression (PR: 1.41, 95% CI: 1.30, 1.52) than Arab Americans with similar ethnic group differences seen for men and women (Table 2).

## Discussion

Arab Americans and Iranian Americans are the two largest subgroups within the MENA ethnic category, but little is known about potential differences in health indicators between these two groups. Our study found significant differences, and some similarities, between Arab Americans and Iranian Americans in the prevalence of risk factors, chronic health conditions, and mental health conditions. Specifically, compared to Arab American men and women, Iranian American men and women had lower age-adjusted prevalence of obesity and current smoking and higher prevalence of hyperlipidemia, prediabetes, anxiety and depression; similar prevalence levels for diabetes and hypertension were observed in the two groups.

Iranian Americans in our study had higher prevalence of hyperlipidemia than Arab Americans. This is consistent with other research that found hyperlipidemia to be higher among Iranian populations than other populations around the world [19]. The high overall prevalence of hyperlipidemia in our study was also in line with a large systematic review of the prevalence of hypercholesterolemia and hypertriglyceridemia in Iran [19]. This systematic review also found that Iranian women had higher rates of dyslipidemia than men, as we found in the oldest age group in our cohort. Arab Americans have been found to have higher prevalence of diabetes than non-Hispanic Whites in multiple settings [20, 21]. The similarity in diabetes and hypertension prevalence among Iranian Americans and Arab Americans and higher prevalence of prediabetes among Iranian Americans, despite significantly higher prevalence of obesity among Arab Americans, suggests that more research is needed to identify dietary and other social, behavioral, or genetic factors that may be differently influencing the development of these risks and chronic conditions in Iranian American adults. Culturally tailored interventions may be needed to reduce cardiometabolic risks in this population.

The Iranian Americans in our study had higher prevalence of diagnosed depression and anxiety than Arab Americans. Middle Eastern populations have been found to have difficulty in discussing feelings of depression due to differences in vocabulary and cultural expectations around sharing these feelings with others [22]. We cannot determine whether the differences observed in our study between Iranian and Arab Americans in prevalence of depression and anxiety reflect a true difference in the experience of these conditions or are a result of differential underdiagnoses of existing conditions due to patients’ reluctance to discuss these types of feelings with their healthcare providers. Little work has been done to characterize the mental health needs of Iranian Americans in the US, although there is some evidence of a stigma associated with mental healthcare access in this population [23]. Differences in mental health may also, in part, be due to differences in experiences of discrimination and bias [24]. If Iranian Americans are experiencing higher levels of discrimination and bias, due to acculturation status or time since immigration, they could be at higher risk for mental health issues than Arab Americans. Alternatively, Iranian Americans may be more comfortable sharing feelings of depression and anxiety with their providers than Arab Americans due to differences in acculturation or culture, although there is no evidence of this in published literature [24, 25]. Immigration related factors, like time since immigration and circumstances related to immigration may also be contributing to differences in mental health outcomes between these two groups [26].

Our study has a number of limitations that should be considered in the interpretation of our findings. First, Iranian Americans included in this analysis had self-described their ethnicity status or indicated Farsi as a native language in their medical records. We may have been unable, therefore, to identify all Iranian Americans in the cohort. There is the potential for selection bias given the fact that individuals with an Iranian/Persian ethnic identification may be different from those without such a designation in their medical records. Second, the majority of Iranian Americans living in California reside in Southern California [27]. Our results may not be generalizable to the entire Iranian American population in California, let alone the US. Third, we could not account for acculturation status, a factor previously shown to impact differences in health outcomes in immigrant groups. While we had information on preferred spoken language, the EHR data did not include length of time lived in the US or other factors known to affect dietary and physical activity practices related to our outcomes of interest. We also lacked information on educational attainment, income, and other social and behavioral determinants of health that may differ between Iranian and Arab American groups.

Despite these limitations, our ability to utilize the electronic health records from a rich and sociodemographically diverse health care delivery system enabled us to demonstrate that there are diverse health needs within the MENA ethnic group. In previous work, we showed that the prevalence of risk factors, chronic and mental health conditions differed between Arab Americans and non-Hispanic Whites in this same cohort [28]. Specifically, Arab American men and women had a higher prevalence of hyperlipidemia and prediabetes than non-Hispanic Whites. Recent evidence suggests that the composition of the non-Hispanic White category may be leading to large within group health differences that are not captured using standard race and ethnicity categories [15]. Given the differential patterns in health found within the MENA group, the additional evidence from this analysis supports further investigations into the diversity within the non-Hispanic White category.

## Conclusions

Our observations are not easily observable using state and national survey data given the ways in which Arab and Iranian Americans are combined within the non-Hispanic White category. This work emphasizes the need to collect granular data on the race and ethnicity of minority Americans in order to help clinicians and public health departments address their health needs and target appropriate interventions to these communities. Further, disaggregating data within the MENA ethnic category, especially in areas with large Arab American and Iranian American representation, may improve our ability to document health disparities and meet the health needs of this growing minority population.

## Data Availability

The Kaiser Permanente Northern California (KPNC) Institutional Review Board has not provided approval for KPNC electronic health record data used for this study to be placed in a public access repository. Additional statistics derived from the data are available from the corresponding author on reasonable request. To request access to data used by this study, researchers should contact the corresponding author, who will then need to get approval for the request from the KPNC Division of Research Data Sharing Workgroup.
